# Primary Breast Tuberculosis Concealed Behind Granulomatous Mastitis

**DOI:** 10.7759/cureus.33447

**Published:** 2023-01-06

**Authors:** Samah Tahri, Siham Hamaz, Houda Bachir, Samia Malki, Younesse Najioui, Amal Bennani, Habiba Alaoui, Khalid Serraj

**Affiliations:** 1 Internal Medicine, Immunohematology and Cellular Therapy Laboratory, University Hospital, Mohammed First University, Faculty of Medicine and Pharmacy, Oujda, MAR; 2 Department of Anatomopathology, University Hospital, Mohammed First University, Faculty of Medicine and Pharmacy, Oujda, MAR

**Keywords:** fine needle aspiration, anti-bacillary drugs, breast carcinoma, mammary tuberculosis, granulomatous mastitis

## Abstract

Granulomatous mastitis is an inflammatory disease that often affects women with a history of breastfeeding. The pathogenesis is still unclear and several factors have been incriminated, such as trauma, metabolic and hormonal disorders, infections, and autoimmunity. This poses a diagnostic issue, given that there are several different diagnoses, particularly carcinomatous mastitis.

We report the case of a 32-year-old woman, with a history of breastfeeding, who presented with inflammatory left breast. The physical examination has objectified a 10/10 cm painless mass and a 3 cm homolateral axillary lymphadenopathy. A sonomammography revealed inflammatory left breast infiltration with multiple collections associated with homolateral axillary lymphadenopathies. A Trucut biopsy was performed, revealing granulomatous mastitis without signs of malignancy. Interferon-gamma measurement and Koch Bacillus (BK) search by polymerase chain reaction (PCR) in the breast collection were all negative. The patient was put on non-specific antibiotics with no response and clinical worsening; therefore, we were obliged to start bacillary treatment. The evolution was marked by a total drought and the disappearance of inflammatory signs within a few weeks.

Mammary tuberculosis poses a diagnostic issue given the difficulty to identify the bacteria in the samples. This is why tuberculosis should never be excluded despite negative results, especially in endemic countries.

## Introduction

Granulomatous mastitis is a rare type of inflammation that is usually benign. It was first documented by Kessler and Wolloch in 1972 [1]. It is a heterogeneous group whose clinical and radiological presentation is varied and non-specific. Mammary carcinoma remains the main differential diagnosis with idiopathic granulomatous mastitis being a diagnosis of exclusion. Primitive mammary tuberculosis is a rare entity. Even in endemic countries, it exhibits incidence levels ranging from 0.25 to 4.5% of total tuberculosis infections [2]. The diagnosis of granulomatous mastitis represents a challenge for clinicians given its scarcity and lack of consensus.

## Case presentation

We report the case of a 32-year-old female patient with a breastfeeding history from the preceding year. No history of diabetes, immunosuppression, or breast cancer in family, and no evidence of tuberculous contact. Two months before her admission, the patient experienced mastodynia and an inflammatory left breast within a context of fever, all while showing no respiratory signs. Clinical examination showed an inflammatory left breast without skin lesions, notably orange skin, retraction, or nipple flow. The palpation revealed a painless 10/10 cm sitting at the junction of inferior quadrants of the left breast with an ipsilateral mobile painless axillary lymph node of 3 cm. The biological assessment showed an inflammatory anemia (Hb: 10.9 g/dl), neutrophilic leukocytosis of 18000 elements/µl, a polyclonal hypergammaglobulinemia in favor of an inflammatory syndrome on the electrophoresis of serum proteins and an elevation of C-reactive protein (CRP) to 160 mg/l.

**Figure 1:**
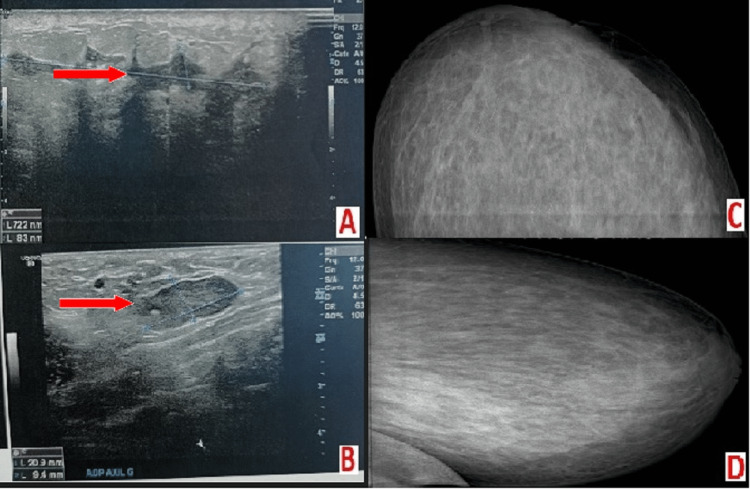
Ultrasound scans. Left breast showing widespread infiltration of the lower quadrants structure (A), associated with homolateral axillary adenopathy of uniform ultrasound ISO structure (B). Mammogram of the left breast, external face (C) and profile (D) showing an opaque veil heterogeneous and little restricted of the lower quadrants.

Taking into account this clinical presentation, the patient received a sonomammography which showed a left-mammary multilocular collection dissecting the infiltrated fibro-glandular tissue that is associated with a necrotic ipsilateral axillary lymph node (Figure 1).

**Figure 2:**
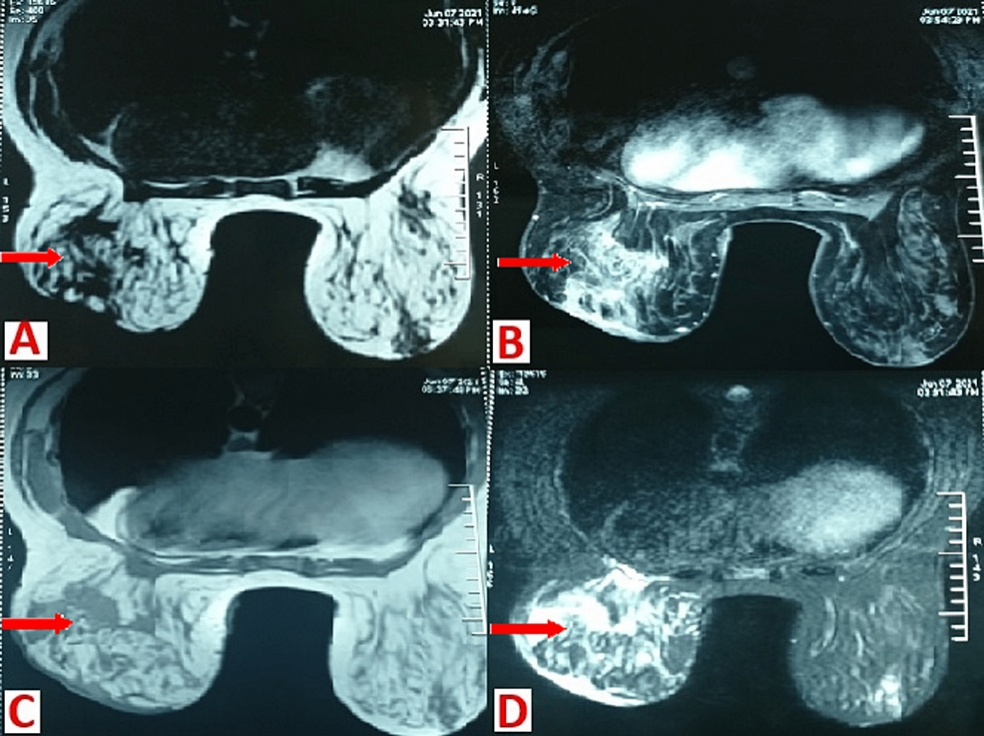
MRI scans. Axial slices of the left breast MRI T1 (A), T2 (B), T1 with injected gadolinium (C), injected T2 (D), showing a minimally limited infiltration of retro-mammillary region and lower quadrants, in ISO T1 signal, discretely heterogeneous T2 hypersignal, raising after injection, infiltrating forward the skin and the areolar plaque and back the deep muscular plane, classified BIRADS IVa.

A magnetic resonance imaging (MRI) scan was conducted to better characterize the lesions and their extensions. The results of the scan revealed mastitis that is of granulomatous origin classified BIRADS IVa (Breast Imaging-Reporting And Data System) according to the American College of Radiology (ACR) with inflammatory axillary lymph nodes (Figure 2).

**Figure 3:**
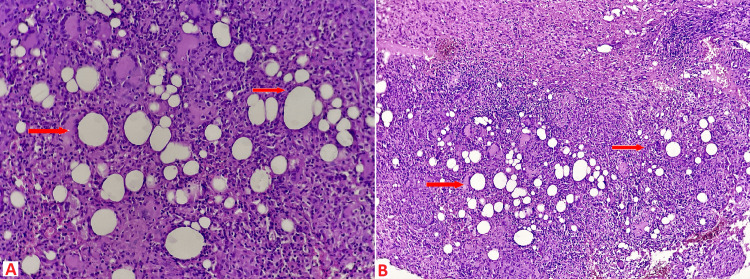
Microphotography. Showing fibrous tissue seat of multiple suppurative lipogranulomas (HE*100) (B), these are composed of central lipid vacuoles bordered with neutrophils and an outer cap of epithelioid histiocytes associated with giant cells Langhans type (HE*200) (A).

A Trucut biopsy was performed, the results of which showed neutrophilic cystic granulomatous mastitis with no signs of tumor proliferation (Figure 3). A puncture in the mammary collection was made to search for *Mycobacterium tuberculosis* through Ziehl-Neelsen staining, culture, and polymerase chain reaction (PCR). The results were negative. Both QuantiFERON-TB Gold and serological brucellosis were also negative. A cervico-thoracoabdominal-pelvic computed tomography scan conducted to search for pulmonary lesions or other deep lymph nodes showed no anomalies. A serological test for human immunodeficiency virus (HIV) also returned negative.

**Figure 4:**
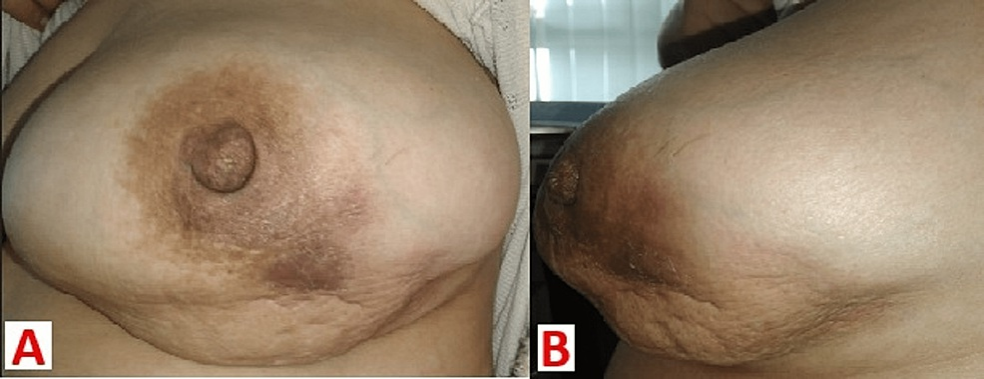
Left breast photography, face (A) and profile (B) showing the disappearance of inflammatory signs a few weeks after starting antibacillal treatment.

Therapeutically, the patient was put under a non-specific antibiotic treatment based on amoxicillin/clavulanic acid at first, then third-generation cephalosporins alongside metronidazole without improvement. This necessitated the commencement of an antituberculous regimen despite the negative report regarding tuberculosis. This time, the development was characterized by a fistula and the release of serous fluids after two weeks. A few weeks after, there was a total dewatering and disappearance of inflammatory signs (Figure 4).

In light of this very adequate response to antituberculous treatment and, after ruling out brucellosis, we retained the diagnostic of tuberculous mastitis and continued the antituberculous therapy that is based on rifampicin, isoniazid, ethambutol, and pyrazinamide for two months, followed with rifampicin and isoniazid for four more months for a total period of six months. The patient has remained clinically stable after one year of follow-up.

## Discussion

The mammary tissue provides resistance against the survival and multiplication of Koch Bacillus, and this accounts for granulomatous mastitis remaining as a rare disease entity even in endemic countries [3]. It often occurs in young women of childbearing age, especially breastfeeding women given that the breast is more vascularized during lactation. Although it is extremely rare, men can also develop granulomatous mastitis [4]. This infection can occur via a direct inoculation of Bacilli through lactiferous ducts, following a spread through a remote site within the body, or rarely by extension of an adjacent thoracic involvement. The incidence of mammary tuberculosis is on the increase in the whole world due to the spread of the infection with HIV [5].

Tewari and Shukla classified mammary tuberculosis into three entities: nodular tuberculosis which occurs in the form of a localized mass that progresses slowly to reach the skin and ulcerate, the disseminated or diffuse form that takes over the full breast with communication between tubers and multiple ulcerations, and lastly, the abscessed form which takes the form of a pyogenic single abscess. The third entity is becoming more frequent [6].

Farrokh et al. and Tulasi et al. have reported cases of the coexistence of carcinoma and mammary tuberculosis. Hence, it is necessary to bear in mind this possibility when dealing with granulomatous mastitis [7,8].

The contribution of mammography remains limited in the diagnosis of mammary tuberculosis because the density of the young women’s mammary tissue makes it difficult to interpret on the one hand; on the other hand, it is impossible, in this review, to differentiate between carcinoma and mammary tuberculosis [6]. The mammography, however, enables to better characterize lesions and to guide the sampling as well as the indispensable biopsies in such cases.

Fine needle aspiration (FNA) with a cytological study confirms the diagnosis of mammary tuberculosis in 75% of cases in the presence of epithelioid granuloma and caseous necrosis since they are highly evocative, though not pathognomonic. The absence of identification of these two findings, however, does not eliminate in any case the diagnosis of tuberculosis [6]. A culture of the aspirated fluid remains the gold standard for searching for *M. tuberculosis* although it is often negative considering the paucibacillary nature of mammary tuberculosis [6]. Microbial identification using PCR is a rapid and specific technique, though of low sensitivity, coupled with the limitation associated with another obstacle, that is, the presence of polymerase inhibitors in approximately 20% of extrapulmonary tuberculosis forms [9].

The 6-month antituberculous treatment provides a good clinical and biological response. It consists of 2-month intensive therapy, made from a classical association of four antituberculosis (isoniazid (INH), rifampin (RMP), pyrazinamide (PZA), and ethambutol (EMB)), followed by a 4-month maintenance phase (INH, RMP) [6]. With some exceptions, surgery is generally not indicated, but in draining abscesses, removing the damaged tissue, as well as in the case of an extended form with no response to antituberculous treatment, or a mammary reconstruction after recovery [6].

## Conclusions

Mammary tuberculous remains a rare condition, yet its occurrence is growing with the extension of HIV infection. It should be suspected in face of all granulomatous mastitis or mammary abscess and especially in absence of response to non-specific antibiotics. The identification of *M. tuberculosis* is exceptional in this localization, its absence, however, should not rule out the diagnosis. The coexistence of tuberculous mastitis and mammary carcinoma is possible; hence the utility of tissue biopsy and the anatomopathological study. Ultimately, the 6-month antituberculous treatment remains the gold standard.
